# *FMR1* Genotype with Autoimmunity-Associated Polycystic Ovary-Like Phenotype and Decreased Pregnancy Chance

**DOI:** 10.1371/journal.pone.0015303

**Published:** 2010-12-16

**Authors:** Norbert Gleicher, Andrea Weghofer, Irene H. Lee, David H. Barad

**Affiliations:** 1 Center for Human Reproduction (CHR) and Foundation for Reproductive Medicine, New York, New York, United States of America; 2 Department of Obstetrics, Gynecology and Reproductive Sciences, Yale University School of Medicine, New Haven, Connecticut, United States of America; 3 Department of Obstetrics and Gynecology, Vienna University School of Medicine, Vienna, Austria; 4 Department of Epidemiology and Social Medicine, Albert Einstein College of Medicine, New York City, New York, United States of America; 5 Department of Obstetrics, Gynecology and Women's Health, Albert Einstein College of Medicine, New York City, New York, United States of America; Bioinformatics Research Centre, Aarhus University, Denmark

## Abstract

The *FMR1* gene partially appears to control ovarian reserve, with a specific ovarian sub-genotype statistically associated with a polycystic ovary (PCO)- like phenotype. Some forms of PCO have been associated with autoimmunity. We, therefore, investigated in multiple regression analyses associations of ovary-specific *FMR1* genotypes with autoimmunity and pregnancy chances (with in vitro fertilization, IVF) in 339 consecutive infertile women (455 IVF cycles), 75 with PCO-like phenotype, adjusted for age, race/ethnicity, medication dosage and number of oocytes retrieved. Patients included 183 (54.0%) with normal (*norm*) and 156 (46%) with heterozygous (*het*) *FMR1* genotypes; 133 (39.2%) demonstrated laboratory evidence of autoimmunity: 51.1% of *het-norm/low*, 38.3% of *norm* and 24.2% *het-norm/high* genotype and sub-genotypes demonstrated autoimmunity (p = 0.003). Prevalence of autoimmunity increased further in PCO-like phenotype patients with *het-norm/low* genotype (83.3%), remained unchanged with *norm* (34.0%) and decreased in *het-norm/high* women (10.0%; P<0.0001). Pregnancy rates were significantly higher with *norm* (38.6%) than *het-norm/low* (22.2%, p = 0.001). *FMR1* sub-genotype *het-norm/low* is strongly associated with autoimmunity and decreased pregnancy chances in IVF, reaffirming the importance of the distal long arm of the X chromosome (*FMR1* maps at Xq27.3) for autoimmunity, ovarian function and, likely, pregnancy chance with IVF.

## Introduction

Recent publications reported associations between number of triple CGG nucleotide repeats on the fragile X mental retardation 1 (*FMR1*) gene and risk towards premature ovarian senescence [1]–[8], leading in milder cases to so-called premature ovarian aging (POA) [2], [9], also called occult primary ovarian insufficiency (OPOI) [10], and at end stage to premature ovarian failure (POF), also called primary ovarian insufficiency (POI) [10]. We recently reported evidence that different *FMR1* genotypes vary in rate of follicle recruitment and, therefore, at least partially, affect functional ovarian reserve, as assessed by anti-Müllerian hormone (AMH) [8].

A normal triple nucleotide (CGG) count range of 26 to 34 repeats (median 30), in respect to ovarian function, allows definition of distinct *FMR1* genotypes, depending on whether both (*normal*), only one (*heterozygous*) or neither (*homozygous*) allele is in normal range [8], [11]. In a small pilot study a *heterozygous-normal/low* (*het-norm/low*) sub-genotype appeared associated with a lean polycystic ovary (PCO)-like phenotype with rapidly depleting ovarian reserve [12].

A PCO-like phenotype is integral to all definitions of the polycystic ovary syndrome (PCOS) [13]. The phenotype, however, solely denotes excessive follicle activity (reflected in high AMH) and, consequently, hyperactivity of ovarian function. PCO and PCOS, therefore, have distinctively different connotations.

Some authors have speculated about a possible autoimmune etiology for selected forms of PCOS [14]–[16]. Others implied such an association when histological demonstrating autoimmune oophoritis with polycystic aspects, accompanied by anti-ovarian antibodies [17]–[19].

At the other end of ovarian function, autoimmunity has been for decades implicated in POF/POI [20]. A very specific phenotype, characterized by a preserved pool of functional follicles, has recently been associated with steroidogenic cell autoimmunity [21]. Hypo-activity is, thus, well documented in association with autoimmunity, while hyperactivity of ovaries is not.

Considering the substantial evidence in support of autoimmune-associated suppression of ovarian function, we have speculated about autoimmune-induced ovarian stimulation in PCOS patients [22], which would mimic the independent duality of, for example, thyroid autoimmunity and function, both etiologically linked, interacting, yet relatively independent of each other [23].

Initial efforts at our center to demonstrate evidence for autoimmune activity in association with PCOS remained unsuccessful (Weghofer A, Gleicher N, unpublished data). We attributed our failure to phenotypical and etiologic variabilities of PCOS, as defined by current Rotterdam criteria [13]. Identifying above noted PCO-like phenotype with close association to the *het-norm/low FMR1* sub*-*genotype offered, however, a unique opportunity to study a clinically homogenous PCO-like patient population. While widely suspected, close genetic markers of PCOS are still lacking [24].

This study confirms close associations between *FMR1* genotypes and ovarian function but for the first time also associates *FMR1* genotypes in infertile women with risk towards autoimmunity and with pregnancy chances in association with in vitro fertilization (IVF).

## Methods

This study involved the retrospective review of medical records. All data utilized were extracted from medical charts and the Center's centralized, confidential electronic research data bank. Patients, at time of initial consultation, sign an informed consent, which permits such utilization of medical records for research purposes as long as the patients' identities remain undisclosed, medical records are untraceable and remain confidential. The Center's Institutional Review Board (IRB) allows such studies under expedited review.

### Patients/cycles

We investigated 339 consecutive female infertility patients who presented to our center for initial diagnostic evaluation, which routinely includes limited genetic and immunologic testing. Amongst those, 75 women qualified as PCO-like phenotypes, based on large numbers of oocytes retrieved (≥12) in first IVF cycles, and/or high anti-Müllerian hormone (AMH, >4.0 ng/mL) at initial evaluations. These cut off values were chosen in consideration of the high prevalence of diminished ovarian reserve in our patient population [9] (see also ovarian reserve tests in Table 1).

**Table 1:** Patient characteristics*.

	Ovarian Phenotype		
	Normal	PCO-like	P-Value
Number	264	75	
Age (years)	38.8±4.8	34.8±4.3	<0.0001
FSH (mIU/MI, 95% CI)	10.3 (9.72–11.03)	7.35 (6.67–8.08)	<0.0001
AMH (ng/mL, 95% CI)	0.55 (0.48–0.62)	2.21 (1.76–2.77)	<0.0001
BMI	22.7±13.5	23.3±13.2	0.74 (N.S.)
Oocytes retrieved (n)	4.8±3.3	17.5±6.7	<0.0001
Embryos transferred (n)	2.4±1.1	2.4±0.8	0.88 (N.S.)
Ethnicity/Race (n/%)			
Caucasian	157 (59.5)	43 (57.3)	
African	33 (12.5)	12 (16.0)	
Asian	38 (14.4)	10 (13.3)	
Middle Eastern	11 (4.2)	2 (2.7)	
Ashkenazi Jewish	15 (5.7)	6 (8.0)	
Other	10 (3.8)	2 (2.7)	
Autoimmunity (n/%)			
No	162 (61.4)	43 (57.3)	N.S.
Yes	102 (38.6)	32 (46.7)	

*Ovarian reserve tests in this table reflect first patient evaluations. IVF outcome data reflect only first IVF cycles.

Data are shown with confidence intervals when log-transformed for analysis and with standard deviations where not. Positive autoimmunity denotes sum of all positive patients, demonstrating at least one abnormality in the immune panel tested (for further details, see Methods section).

The 339 patients underwent 455 IVF cycles, 116 thus representing repeat attempts. As Appendix S1, however, demonstrates, adding a covariate for number of IVF cycles experienced by each patient does in our patient population not affect the findings of an analysis based on per patient outcomes.

Genetic testing includes an assessment of triple CGG nucleotide repeats on the *FMR1* gene, as reported before [5]–[8]. We previously also reported that, in regards to ovarian function, 26 to 34 repeats represent a normal range (median 30) [8], and counts below and above denote risk towards POA/OPOI [6]. Based on this normal range, *normal* (*norm*), *heterozygous* (*het*) and *homozygous* (*hom*) *FMR1* genotypes can be described, which are reflective of distinct ovarian aging patterns [8]. They are defined by both alleles in normal range (*norm*), either one outside of range (*het*) or both alleles outside of range (*hom*). A *het* patient, in turn, can be either *het-norm/low* (<26 repeats) or *het-norm/high* (>34 repeats), while *hom* patients may be either *high/high*, *high*/*low* or *low/low*.

The *het-norm/low FMR1* genotype has in a longitudinal and cross-sectional study been associated with very high ovarian reserve (based on AMH) at young ages, which, however, in the early 30 s, quickly depletes, resulting in rapid AMH declines at relatively young ages and relatively diminished age-dependent ovarian reserve thereafter [12]. Women with this genotype after ages 32–33 years, therefore, lose their PCO-like phenotype and, without *FMR1* evaluations, based on AMH levels alone, would be perceived as either normal or suffering from prematurely diminished ovarian reserve.

Immunological testing involves, as previously reported [25], total immunoglobulin levels (IgG, IgM, IgA), antinuclear, anti-phospholipid and anti-thyroid antibody panels, as well as anti-ovarian and anti-adrenal antibodies. We previously demonstrated that this panel of immunological tests is sufficient in identifying even subclinical levels of autoimmunity that predisposes towards POA/OPOI [25], [26]. In utilizing this established screening method, a patient was defined as autoimmune-negative with absence of any abnormality and was considered autoimmune-positive with presence of even one, fully recognizing that such a definition increases sensitivity at expense of specificity and, therefore, weakens the discovery of potentially existing associations with autoimmunity. This definition of autoimmunity, therefore, consciously biases results against positive association and, thus, strengthens any detected associations.

IVF cycles were conducted in routine fashion. In principle, only two ovarian stimulation protocols were utilized: women with normal ovarian reserve were down-regulated with a gonadotropin releasing hormone agonist and stimulated with maximally 300 IU of gonadotropins daily. Patients with diminished ovarian reserve received a micro-dose agonist protocol with 450 to 600 IU of gonadotropins daily. Hormone assays for follicle stimulating hormone (FSH), estradiol and AMH were run in house as previously reported [27].

### Statistical Analysis

Patient characteristics and IVF cycle outcomes were evaluated in association with *FMR1* genotypes and autoimmune status, as defined above. PCO-like phenotypes were distributed in 62 percent as *norm*, 25 percent as *het-norm/low* and 13 percent as *het-norm/high* and represented 22 percent of all patients. Women with normal ovarian phenotype were in same order distributed at 52, 28 and 20 percent, respectively and represented 78 percent of all patients.

This distribution corresponds to an effect size, w, of 0.091 and, equivalently, to a contingency coefficient (C) as well as a Cramer's phi coefficient (phi) of 0.091. With a sample size of 339 the study, thus, had only a power of 30.3% to yield statistically significant results. Assuming continuation of in this study observed proportions, a study with identical effect size (w = 0.091) would require a sample size of 1,164 patients to achieve an 80 percent power to detect a significant *alpha* (0.05, two-tailed).

Univariate comparison between women with PCO-like phenotype and control was performed using Chi Square and analysis of variance as appropriate. Variables in which the distribution of data did not conform to normality were first log transformed for analysis and then converted back to standard units for presentation. Where cycles served as study units, odds ratios were compared. Continuous variables are presented as either mean ± standard deviation (SD) or mean and 95 percent confidence interval (95% CI), as appropriate.

We constructed a general linear model with logit link function, testing the association of the PCO-like phenotype with *FMR1*, and autoimmunity and also for pregnancy. General linear models were run on SAS version 9.2 (GENMOD module).

## Results

Table 1 summarizes patient characteristics for all 339 women, amongst those 264 (77.9%) representing a normal, non-PCO-like ovarian phenotype and 75 (22.1%) the defined PCO-like phenotype. Women with PCO were younger (34.8±4.3 vs. 38.8±4.8 years, P<0.0001), had lower FSH (8.0±4.0 vs. 11.9±6.8 mIU/mL, P<0.0001) and higher AMH levels (3.2±2.6 vs. 0.8±0.7 ng/mL P<0.0001) and produced larger oocyte yields (17.5±5.6 vs. 4.8±3.3, P<0.0001). Otherwise, the two groups did not differ significantly, including in ethnic/racial distribution and BMI, confirming the non-obese nature of the here investigated PCO-like phenotype.

### *FMR1* Genotypes

Table 2 summarizes *FMR1* genotype distribution patterns in women with apparently normal and PCO-like ovaries. Amongst 264 women with normal ovaries 137(51.9%) demonstrated a *norm FMR1* genotype; the remaining 127 (48.1%) were *het* (there were no *hom* patients in the study population), 52 (19.7%) were *het-norm/high* and 75 (28.4%) *het-norm/low*. This distribution did not significantly differ from the *FMR1* genotype distribution amongst the 75 women with PCO-like phenotype, where *norm* were 46 (61.3%), *het-norm/high* 10 (13.3%) and *het-norm/low* 19 (25.3%). The previously noted age-dependency of the definition of the PCO-like phenotype [12] mandates caution in interpreting these results.

**Table 2:** *FMR1* genotype distribution*.

Phenotype	Norm	Het-Norm/High	Het-Norm/Low
PCO-like	46 (61.3)	10 (13.3)	19 (25.3)
Normal	137 (51.9)	52 (19.7)	75 (28.4)
Total	183 (54.0)	62 (18.3)	94 (27.7)

*Chi-Square 2.46, df  = 2.0, P = 0.29.

### Autoimmunity

Distribution of autoimmunity also did not differ between both patient groups (Table 1): In women with normal ovaries 162/264 (61.4%) showed no evidence of autoimmunity and 102 (38.6%) did, while in the PCO-like cohort 43 (57.3%) did not and 32 (46.7%) did.

**Figure 1:**
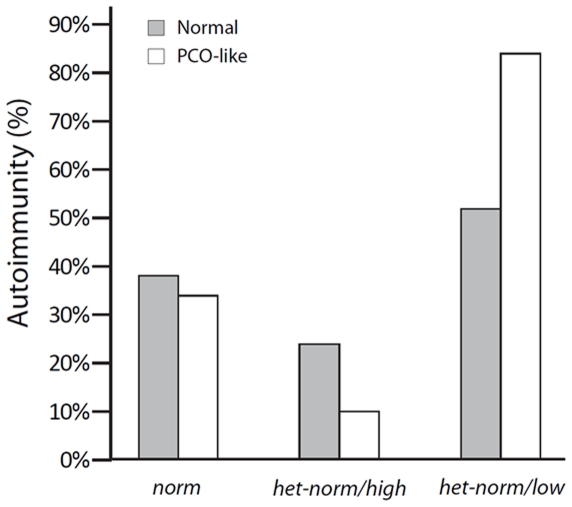
Prevalence of autoimmunity in reference to FMR1 genotype. The prevalence of autoimmunity was in both patient groups the highest with *het-norm/low FMR1* genotype and the lowest with *het-norm/high* genotype. This pattern, however, intensified in women with PCO-like phenotype. Gray bars represent women with normal ovarian reserve; white bars represent the PCO-like phenotype.

If autoimmunity was, however, assessed in reference to *FMR1* genotype (Figure 1), the *het-norm/low* genotype was most frequently associated with autoimmunity (51.1%), followed by *norm* patients (38.3%) and *het-norm/high* women (24.2%), a statistically significant difference in distribution (p = 0.003). These differences in distribution further strengthened in women with PCO-like phenotype: *het-norm/low* women demonstrated autoimmunity in 83.3 percent of cases, while *het-norm/high* genotypes in only 10.0 percent, almost categorically differentiating between these two *het* genotypes, while *norm* women held a middle ground with 34.0 percent prevalence (P<0.0001).

### IVF Pregnancy Rates

**Figure 2:**
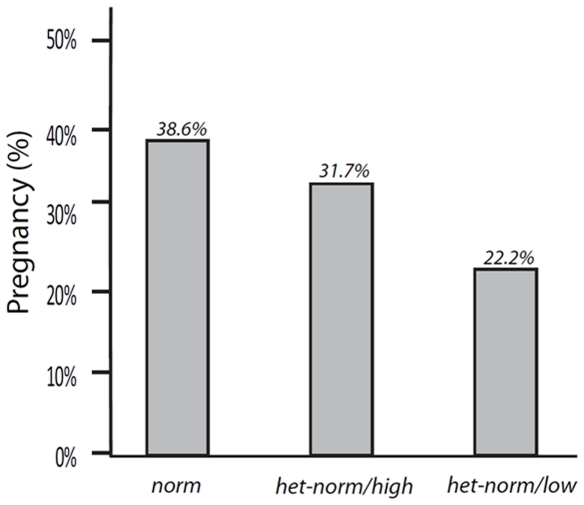
Pregnancy rates in IVF based on *FMR1* genotype. Pregnancy rates were the highest with *norm FMR1* genotype and the lowest with *het-norm/low* genotype.

Pregnancy outcomes in 455 consecutive IVF cycles are summarized in Figure 2. As the figure demonstrates, *norm* women experienced the highest pregnancy rates (38.6%), a rate significantly higher than in *het-norm/low* patients [22.2%; OR 0.84 (95% CI 0.74 to 0.96; Wald 6.9; df = 1; P = 0.009)]. Women with *het-norm/high* genotype had intermediate pregnancy rates at 31.7 percent. Adjustment for age maintained the disadvantage in pregnancy rate *for het-norm/low* women (OR 0.43; 0.22 to 0.86, p = 0.017).

## Discussion

This study demonstrates that in consecutive patients presenting for infertility treatments the prevalence of autoimmunity varies significantly with *FMR1* genotype, with *het-norm/low* presenting with most and *het-norm/high* with least autoimmunity. This distribution is further strengthened in infertile women with the lean PCO-like phenotype, we previously described in a pilot study associated with the *het-norm/low FMR1* genotype and with relatively rapidly depleting ovarian reserve [12]. Such patients almost guarantee positive autoimmune laboratory findings (83.3% prevalence, Figure 1), while a *het-norm/high* genotype is practically protective against autoimmunity (10.0% prevalence). Women with *norm FMR1* genotype in both patient populations take up a middle ground with 38.3% and 34.0% prevalence, respectively.

The close association between the *het-norm/low genotype* and autoimmunity is further supported by the fact that the PCO-like phenotype was significantly younger (P <0.0001, Table 1), while autoimmunity actually increases in prevalence with advancing female age [28]. PCO-like phenotypes, thus, demonstrated significantly more autoimmunity, despite significantly younger ages.

The statistical clarity of here reported results is, however, especially remarkable, considering that patient selection criteria in this study strongly biased against discovery of such statistical associations. As already previously noted, the definition of positive autoimmunity consciously was based on improving sensitivity at the expense of specificity. Women defined as autoimmune, therefore, likely included a few without real polyclonal autoimmune activation.

Even more significantly, however, we noted earlier the time line for premature declines in ovarian reserve in women with the *het-norm/low FMR1* genotype [12]. Even though the here investigated group of patients with PCO-like phenotype were significantly younger (P<0.0001, Table 1), it appears likely that older women with apparently normal ovarian phenotype must include at least some who at younger ages actually did demonstrate a PCO-like phenotypes.

Patients with PCO-like phenotype, in this study defined by 12 or more oocytes retrieved and/or an AMH above 4.0 ng/mL, represented 75 (22.1%) of all patients investigated. The definition of the PCO-like phenotype in this study was purely clinical, meant to identify a patient population with disproportionally high ovarian reserve, as documented by high oocyte yields and AMH values. Considering that the here investigated patient population included, as also previously reported [9], a disproportionate number of women with significantly diminished ovarian reserve (confirmed by elevated FSH, low AMH and oocyte yields in women with normal ovarian phenotype, Table 1), here chosen cut offs, defining a PCO-like phenotype for study purposes, appear appropriate. Twelve or more oocytes in such patients are above expected averages, as even women under age 35 years at our center produce only an average of 8.2±5.8 oocytes [29]. Similarly, an AMH above 4.0 ng/mL exceeds the 95% confidence interval (CI) of AMH levels at our center in women as young as age 26 years [30].

Here reported autoimmune laboratory findings in slightly above one third of women correspond well to prevalence numbers for infertility populations, reported in the literature [31], [32]. This not only validates the selected study population but also reaffirms the immune profile used in this, and prior studies [25], [26], to define presence of subclinical levels of autoimmunity. Though not reaching significance, prevalence of autoimmunity further increased (38.6% to 46.7%) from normal ovarian to PCO-like phenotypes. As further discussed below, this finding appears primarily the consequence of the strong association between *het-norm/low FMR1* genotype and autoimmunity.

To define autoimmunity at subclinical levels is difficult to impossible, and is the reason why clinical diagnoses of autoimmune conditions in prodromal stages often are difficult [28]. Autoimmunity is, however, typically associated with a polyclonal activation of the immune system, which can be detected by broadly based laboratory evaluations [33], [34]. While such screens are not specific enough for diagnoses of autoimmune diseases, they appear sensitive enough in defining evidence of autoimmune activity [25], [26].

This study, once again, reaffirms this by demonstrating a surprisingly close association between autoimmunity and the *het-norm/low FMR1* genotype. We already previously associated the *het-norm/low* genotype in a pilot study with a PCO-like phenotype, with rapidly depleting ovarian reserve [12]. Women with this genotype present at young ages with a PCO phenotype. Because of rapid follicle depletion (i.e., rapidly diminishing ovarian reserve), they then at older ages demonstrate normal to abnormally low AMH levels, reflecting relative or outright diminished ovarian reserve.

Since the *FMR1* gene appears closely involved with regulation of follicle recruitment and, therefore, ovarian reserve [1]–[8], the association between *het-norm/low FMR1* genotype and PCO-like phenotype does not surprise. The extremely close association between *het-norm/low* genotype and autoimmunity was, however, completely unanticipated. This association appears, indeed, so close that in a PCO-like population a *het-norm/low* genotype virtually predicts autoimmunity, while women with the *het-norm/high* genotype appear protected from autoimmunity.

These associations also translate into clinical significance for infertile women since the *FMR1* genotype appears predictive of pregnancy chances with IVF. Women experience best pregnancy chances with *norm*, intermediate with *het-norm/high* and lowest rates with *het-norm/low* genotypes (Figure 2).

Whether a PCO-like phenotype, alone, affects pregnancy chances in IVF has remained controversial [35]. Lower [36] and similar [37] pregnancy rates have been suggested in PCOS in comparison to other infertile patients undergoing IVF. Multiple underlying etiologies for PCOS [13], different ovarian stimulation protocols and variability in genetic definitions of study populations easily explain such discrepancies.

The same kind of controversy surrounds the association of autoimmunity and pregnancy success in IVF. Many authorities have categorically denied an association [38]–[40], while others have pointed at considerable evidence [41], [42]. Here presented data suggest a possible explanation for these contradictory opinions since, like PCOS, most studies on autoimmunity have been performed in genetically and etiologically heterogenic patient populations. At least in a genetically homogenous population of women with the *het-norm/low FMR1* genotype, autoimmunity, indeed, appears negatively associated with pregnancy chances in IVF.

Autoimmunity is abnormally high in practically all X-linked disorders [43]. If defective, a MHC-paralogue on the long arm of the X chromosome renders individuals immunologically less efficient [44]. With the *FMR1* gene mapping to Xq27.3 [45], it appears to occupy the cross roads between ovarian function (ovarian recruitment and ovarian reserve) and autoimmunity [43], [44], [46].

Both autoimmunity and abnormalities in ovarian reserve are closely associated with X chromosome defects: A good example is Turner syndrome, in which Xq21 terminal deletions are common, often large and characterized by primary as well as secondary amenorrhea [46], [47]. Xq21 or further distal deletions usually present with secondary amenorrhea [47], a classical clinical presentation of premutation range *FMR1* (fragile X) carriers who present with POF/POI [10], [48]. In contrast Turner syndrome with normal fertility generally involves more proximal Xq deletions [49].

POF/POI, indeed, demonstrates a 4MB locus exactly at Xq27-q28 [46], with the *FMR1* gene mapping to Xq27.3 [45]. Small deletions in Xq27-q28 have variable phenotypes, some with early menopause but are usually able to reproduce until experiencing full POF/POI [46]. Similar correlations with ovarian function are also observed in balanced translocations, where only Xq23-q27 deletions are associated with POF/POI [46].

Turner syndrome is not only characterized by above noted abnormalities in ovarian function but, like most X-linked disorders, also by excessive autoimmunity. Both autoantibodies and autoimmune diseases are significantly increased [44]. The close association between X-linked disorders and autoimmunity led to the suggestion that the latter may be the consequence of genes and/or mutations on the long arm of the X chromosome [43], potentially explaining the increased prevalence of autoimmunity in women in comparison to males [50].

A PCO-like phenotype with strongly associated autoimmunity and specific *FMR1* genotype (*het-norm/low*), and the protective effect of another *FMR1* genotype (*het-norm/high*), support the notion we made earlier [8] that the *FMR1* gene plays a role in regulating ovarian reserve. Now it appears that the same gene may also be involved in determining risk/protection of/from autoimmunity.

These observations also raise the intriguing possibility that a PCO-like phenotype may be associated with an autoimmune etiology. This has previously been suggested [14]–[19], [22] but, in contrast to cases of POF/POI [20], [21], PCO and/or PCOS have never been established as autoimmune in nature. Like POF/POI in its various forms [10], [25], [26], [51], PCOS is multifactorial in etiology [13], with PCO being a unifying phenotypical presentation of an, otherwise, still very controversial syndrome [52].

Only very recently, Hefter Frischmuth and associates reported serological evidence for elevated autoimmunity in women with PCOS [16]. The authors, like also in this study, investigated non-organ specific (antihistone and anti-dsDNA) antibodies. Another Austrian group, however, even more recently, reported a statistical association between levels of anti-thyroid peroxidase antibodies and treatment response in women with PCOS, suggesting that organ specific thyroid antibodies may also be associated with PCOS [53]. Thyroid autoimmunity, of course, is well known to be closely associated with ovarian autoimmunity [20].

These reports and here presented data support the hypothesis that, like other endocrine organs, ovaries may be subject to suppressive and stimulatory autoimmune influences, possibly mediated by autoantibodies, a hypothesis we previously proposed after Baroni and associates reported stimulatory antibodies (to platelet derived growth factor) in systemic sclerosis [54]. Building on their findings, we speculated that “functional” autoantibodies may represent a universal paradigm of autoimmunity [22].

In regards to ovarian function such a concept would suggest inhibitory autoantibodies as cause of selected cases of POA/OCPOI and POF/POI, as recently suggested by La Marca and associates [21], and stimulatory autoantibodies as promoters of follicular activity, resulting in at least one PCO-like phenotype. Here reported strong positive and negative associations with both *het*-*FMR1* genotypes further suggest a possible role of the *FMR1* gene in regulation of these “functional” autoantibodies and, therefore, possibly, in contributing to the well established higher prevalence of autoimmunity in females in comparison to males [42].

Despite the substantial size of the here reported population of infertile women, this study's principal weakness lies in its relatively limited power. As, however, previously noted, to achieve 80 percent power for a significant *alpha* under observe proportions, close to 1,200 patients would be required. While we are continuing to accumulate patients, this is a difficult goal to obtain in a single center study.

## Supporting Information

Appendix S1 Pregnancy analysis of only 339 1^st^ IVF cycles^*^.
(DOC)
