# Supplementary material for: FMR1 Genotype with Autoimmunity-Associated Polycystic Ovary-Like Phenotype and Decreased Pregnancy Chance
Source: PLoS One. 2010 Dec 16;5(12):e15303. doi: 10.1371/journal.pone.0015303 (PMC3002956; doi:10.1371/journal.pone.0015303)
Supplement: Appendix S1 — Pregnancy analysis of only 339 1st IVF cycles*. (DOC) [file pone.0015303.s001.doc]

Appendix I. Pregnancy analysis of only 339 1^st^ IVF cycles*

|  | Norm | Het-norm/low | Het-norm/high |
| --- | --- | --- | --- |
| Pregnant [n (%)] | 54 (29.5) | 14 (14.9) | 15 (24.2) |
| Not pregnant | 129 (70.5) | 80 (85.1) | 47 (75.8) |

*Chi-Square 7.17; df = 7.17; P=0.028
